# Foreign Body Aspiration in Pregnancy

**DOI:** 10.1155/2012/890106

**Published:** 2012-11-25

**Authors:** Andrew L. Atkinson, Joseph Canterino

**Affiliations:** Department of Obstetrics and Gynecology, Jersey Shore University Medical Center, Neptune, NJ 07753, USA

## Abstract

A 24-year-old morbidly obese African American gravida 1, with a history of severe asthma complicated by multiple inpatient admissions, presents at 30 weeks gestation with a foreign body in her left main stem bronchus. After a failed bronchoscopy postpartum, the patient slipped into respiratory failure and was subsequently intubated, spending two weeks in the intensive care unit. After two more attempts of trying to retrieve the foreign object from her lung via bronchoscopy, she eventually contracted a postobstructive pneumonia and underwent a left lower lung lobectomy for curative treatment.

## 1. Introduction

Foreign body aspiration in the pregnant patient is an area of medicine that has relatively little published material describing management or treatment. Asthma as well as foreign body aspiration in the pregnant as well as the nonpregnant patient can cause varying levels of hypoxia. Hypoxemia in the pregnant patient may contribute to low birth weight, preeclampsia, congenital malformations, spontaneous abortions, and placenta previa in oxygen deprived females [1]. Hospitalizations have been reported to occur in 1.6% and emergency room visits in 12.6% of pregnant asthmatic patients alone [2]. Concerning the patient that we are presenting, despite an already poor baseline respiratory function secondary to her poorly controlled asthma, it was found in the mid 2nd trimester that she had aspirated a foreign body which complicated management further. 

## 2. Case Report

**Figure 1:**
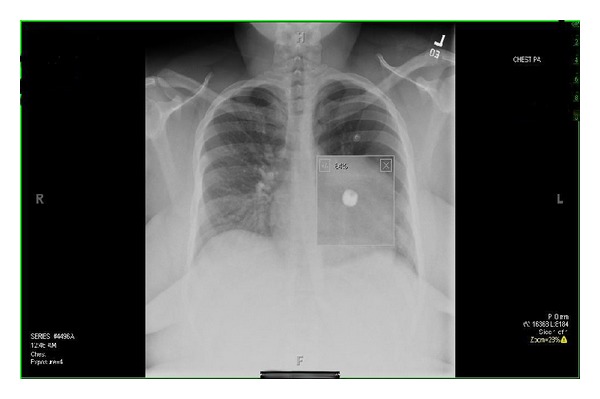
Chest X-ray showing magnified image of the “ball” portion of the tongue piercing in the left main stem bronchus.

Twenty-four-year-old morbidly obese African American gravida 1, whose pregnancy was complicated by poorly controlled asthma, was subsequently admitted fourteen times over the duration of her pregnancy for medical stabilization, seven of those admissions being in the first trimester alone. At 30 weeks gestation the patient was admitted for asthma exacerbation, and a chest X-ray was performed. A well-circumscribed 0.64 × 0.59 × 0.64 radio-opaque lung nodule was identified on a posterior/anterior view (Figure 1). When the patient was initially questioned about the foreign body in her left main stem bronchus, the patient stated that she had swallowed her tongue piercing. The patient went on to say that this was her third tongue piercing in a 15-month time span; all others were swallowed incidentally as well.

Given the patients' poor respiratory status now complicated by an aspirated foreign body, pulmonology was placed on consult to assist with management. The risks versus benefits of bronchoscopy in the antepartum period were discussed in detail with the patient as well as a physician team that was involved in the care of this patient. It was decided that the combination of poorly controlled asthma and anesthesia is needed for the proposed bronchoscopy; the risk of respiratory failure was too high for the mother who was only at 33 weeks gestation at that time. Surveillance of the fetus was done with serial ultrasounds on a biweekly basis and nonstress tests were done on a daily basis. Estimated fetal weight was consistently found to be at the 40th percentile adjusted for gestational age, with no signs of fetal distress on daily fetal testing. Eventually at 35 weeks the patient was induced for maternal indications due to a deteriorating respiratory status and went on to deliver vaginally a liveborn male infant without complications. The placenta that was delivered was noted to have very large calcifications (some as large as three to four centimeters in diameter), which confirmed the degree of hypoxia that occurred during this pregnancy.

**Figure 2:**
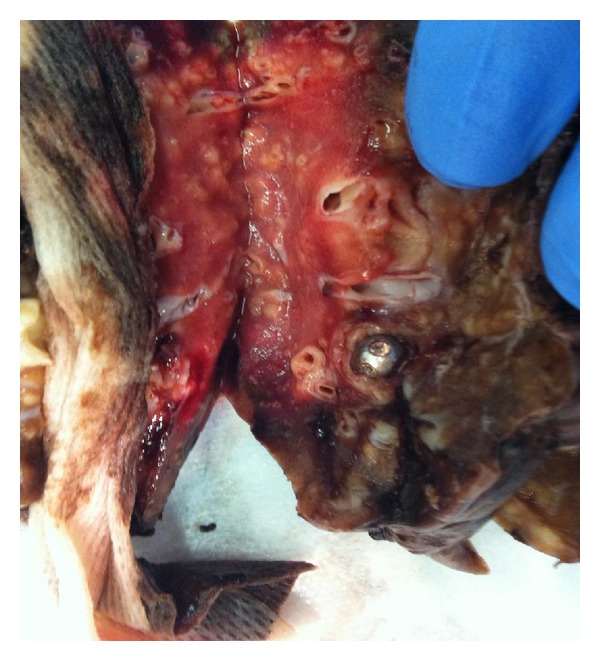
Gross image of the excised lower lobe of lung with foreign object imbedded in a terminal bronchiole. Note the necrotic lung tissue distal to the obstructed bronchiole.

 Following childbirth the patient was brought to the endoscopy suite for a diagnostic as well as a therapeutic bronchoscopy. Of note, the patient's asthma was still poorly controlled despite delivering her child one week earlier. The patients' asthma continued to show no improvement despite treatment with high-dose steroids as well as routine beta 2 agonist therapy. It was decided to go ahead with removal of the foreign body in the left lung with the hope that her respiratory status would improve with expulsion. A flexible video bronchoscope was performed because of the distal location of the tongue piercing; her bronchial tissue was noted to be very friable and bled easily when grazed by the bronchoscope. The bronchoscopy failed to retrieve the foreign body, and as a postprocedure the patient was intubated by the anesthesia team because of extremely low oxygen saturations postprocedure and subsequently spiraled down into respiratory failure as feared earlier by the pulmonology team. An upright chest X-ray post procedure was done and the left lower base of the lung was noted to be completely clouded over; a supine X-ray was done as well; clear bases were seen bilaterally suggesting a gravitational shift of blood pooling in the left lung. The patient went on to spend two weeks intubated in the intensive care unit (ICU) with the diagnosis of acute respiratory failure, complicated by a postobstructive pneumonia secondary to the foreign body. Two more bronchoscopies were attempted to retrieve the tongue piercing, once again using flexible bronchoscopy, but both ended in failure. Despite aggressive antibiotic treatment, her pneumonia was not improving. The patient was finally taken to the operating room, and a left lower lung lobectomy was performed (Figure 2). 

Postoperatively the patient returned to the ICU extubated, only on 2 liters via nasal cannula. The patient was discharged from the ICU on postoperative day 2, and spent five days on a medical/surgical step-down unit before being discharged to a rehabilitation facility. When discharged from the hospital, the patient was off all antibiotics and was only on a steroid taper as well her asthma medication regimen she was on prior to getting pregnant. After a two-week stay in an inpatient rehabilitation facility as well as a five-day stay at an inpatient psychiatric facility for major depression, the patient went on to make a full recovery. 

## 3. Discussion

We present a very unusual case of foreign body aspiration during pregnancy, complicated by respiratory failure that eventually leads to a complete lobectomy of the left lung. In the gravid oxygen-starved patient, fetal growth should be monitored closely. There is likely to be a link between changes in maternal hypoxemia and the increased risk of poor pregnancy outcomes. This has been suggested by several studies where worsening of oxygenation status was associated with reduced fetal birth weight [3]. In the brain, hypoxia can produce temporary brain dysfunction or permanent brain injury, depending on the duration, degree of oxygen deprivation, and the age of the fetus [4]. In regards to bronchoscopy in the pregnant patient, risk in the pregnant patient versus the nonpregnant patient is relatively unknown. In the nonpregnant patient, the reported mortality rate of bronchoscopy is 0.01% to 0.04%, with major complications occurring 0.08–.5% of the time [5]. Risk factors for maternal hypoxia from bronchoscopy include medications given for sedation, vagally mediated bronchospasm, and pulmonary aspiration [6]. Although it may be assumed that fetal desaturation is not likely in the absence of maternal respiratory depression, it is reasonable to maintain higher maternal oxygen saturations (e.g., >96%) than would normally be tolerated routinely in nonpregnant patient (>90%) [7]. Bronchoscopy still continues to be regarded as a safe procedure in the pregnant patient and is still the gold standard procedure of choice for extraction of a foreign body in the lung of the pregnant patient. The patient was not a typical case of foreign body aspiration, and because of her underlying lung disease, a bronchoscopy would have put her and her fetus at too much risk if she did spiral down into respiratory failure in the antepartum period. As with all invasive procedures in the pregnant patient the physician has to be cognizant of the risks versus benefits when treating not only the mother but the growing fetus in utero.
